# Comparing U.S. Injury Death Estimates from GBD 2015 and CDC WONDER

**DOI:** 10.3390/ijerph15010087

**Published:** 2018-01-07

**Authors:** Yue Wu, Xunjie Cheng, Peishan Ning, Peixia Cheng, David C. Schwebel, Guoqing Hu

**Affiliations:** 1 Department of Environmental and Occupational Health, Xiangya School of Public Health, Central South University, 110 Xiangya Road, Changsha 410078, China; wuyue7802@csu.edu.cn; 2 Department of Epidemiology and Health Statistics, Xiangya School of Public Health, Central South University, 110 Xiangya Road, Changsha 410078, China; chengxunjie163@163.com (X.C.); ningpeishan@csu.edu.cn (P.N.); chengpeixia92@163.com (P.C.); 3 Department of Psychology, University of Alabama at Birmingham, Birmingham, AL 35294, USA; schwebel@uab.edu

**Keywords:** injury, death, compare

## Abstract

*Objective*: The purpose of the present study was to examine consistency in injury death statistics from the United States CDC Wide-ranging Online Data for Epidemiologic Research (CDC WONDER) with those from GBD 2015 estimates. *Methods*: Differences in deaths and the percent difference in deaths between GBD 2015 and CDC WONDER were assessed, as were changes in deaths between 2000 and 2015 for the two datasets. *Results*: From 2000 to 2015, GBD 2015 estimates for the U.S. injury deaths were somewhat higher than CDC WONDER estimates in most categories, with the exception of deaths from falls and from forces of nature, war, and legal intervention in 2015. Encouragingly, the difference in total injury deaths between the two data sources narrowed from 44,897 (percent difference in deaths = 41%) in 2000 to 34,877 (percent difference in deaths = 25%) in 2015. Differences in deaths and percent difference in deaths between the two data sources varied greatly across injury cause and over the assessment years. The two data sources present consistent changes in direction from 2000 to 2015 for all injury causes except for forces of nature, war, and legal intervention, and adverse effects of medical treatment. *Conclusions*: We conclude that further studies are warranted to interpret the inconsistencies in data and develop estimation approaches that increase the consistency of the two datasets.

## 1. Introduction

If they are consistent, multiple data sources estimating health outcomes provide stronger evidence than a single data source. The Global Burden of Disease (GBD) estimates offer a widely-used free-access data source with country-specific estimates of fatal and nonfatal burden from diseases and injuries in 195 countries/territories [1]. GBD estimates are increasingly being applied to support health decision-making at global, national, and subnational levels [2,3,4]. The GBD adopts multiple complex models to produce health estimates by adjusting for the impact of unavailable data and poor reporting (e.g., underreporting, misclassification, garbage codes) on country-specific estimates [2,3,4,5]. To evaluate the influence of GBD models in outcomes, comparisons of GBD estimates to other presumably-somewhat reliable data sources are warranted.

We focused our analysis on injury outcomes, which offer an objective health outcome that is the leading cause of death for U.S. citizens between the ages of 1 and 44 years. We focused our analysis on the United States because it is the only nation we identified that is reputed to have accurate health data and that provides publicly available, online, and code-specific injury mortality data in English or Chinese to allow for comparisons using the injury classification system defined by the GBD group. Our study compared injury death statistics from the United States (U.S.) CDC Wide-ranging Online Data for Epidemiologic Research (CDC WONDER) with those from GBD 2015 estimates.

## 2. Materials and Methods

### 2.1. Ethical Issues

This study uses anonymous open-access data and does not involve personal information from individuals. The research was approved by the Ethic committee of Xiangya School of Public Health, Central South University, China on 27 December 2016 (no. XYGW-2016-25).

### 2.2. Data Sources

Death data were retrieved through the GBD online visualization tool and the CDC WONDER online databases [1,6].

The cause of death database of the GBD 2015 update contains seven types of data sources: vital registration, verbal autopsy, cancer registry, police records, sibling history, surveillance, and survey/census [3]. A majority of the cause of death data is vital registration data obtained from either the WHO Mortality Database or mortality databases operated by official offices in individual countries. For the United States, mortality data listed in the WHO Mortality Database are the same as those included in the U.S. CDC WONDER database. Each cause of death is coded directly to the most detailed cause of death in the International Classification of Diseases (ICD). To account for situations where there is poor cause of death detail for coding, select cause groups are disaggregated (e.g., liver cancer and cerebrovascular disease (Level 3); liver cancer due to hepatitis C and ischemic stroke (Level 4)). GBD researchers also estimate proportions of deaths from nearby countries within super-regions that may have similar cause of death trends due to their geographic, cultural, and environmental similarities. Data from countries without extensive vital registration coverage are excluded from models for countries with this coverage to avoid inflation of uncertainty.

One challenge for the GBD investigators examining cause of death data is “garbage codes”, which are defined as codes to which deaths are assigned that cannot or should not be considered as the underlying cause of death. These might include exposure to unspecified factors (X59) and undetermined intent codes (Y10–Y34) [3]. To cope with this challenge, the GBD group developed a series of models to correct the problems, including the redistribution of deaths recorded as garbage codes [5]. The GBD determined 95% uncertainty intervals (95% UI) by creating 1000 draws for the final ensemble. The 95% UI is created from the 0.025 and 0.975 quantiles of the draws.

The CDC WONDER mortality database is based on annual mortality data compiled by the National Center for Health Statistics (NCHS) at CDC, which provides counts of underlying cause of death by ICD Code as derived from death certificates [7]. It is believed that more than 99% of the births and deaths occurring in the U.S. are registered [7]. A series of quality control procedures are carried out to ensure validity of the data, such as collecting a sample of 100–175 records per month from each registration area to monitor the quality of coding medical items [7]. The number of deaths reported for an area represent complete counts of such events and, therefore, are not subject to sampling error [7]. However, when the figures are used for analytical purposes, the number of deaths that actually occurred may be considered as one of a large series of possible results that could have arisen under the same circumstances [8]. The probable range of values may be estimated from the actual figures according to certain statistical assumptions. Thus, estimates of relative standard errors (RSE)—a measure of variability—are calculated according to Poisson distribution and expressed as 95% confidence intervals [7,8]. Mortality data included in CDC WONDER are regarded as official statistics and have been widely applied in health research [9,10,11,12].

### 2.3. Data Analysis

Both sources provide fatal injury data in the U.S. for 2000, 2005, 2010, and 2015. We used definitions, classifications of injury and associated codes from the 10th revision of International Classification of Disease, as defined by the GBD study (Table 1) [3]. To eliminate any impact of selecting a standard population, we used crude death data rather than age-standardized data. 

## 3. Results

**Figure 1:**
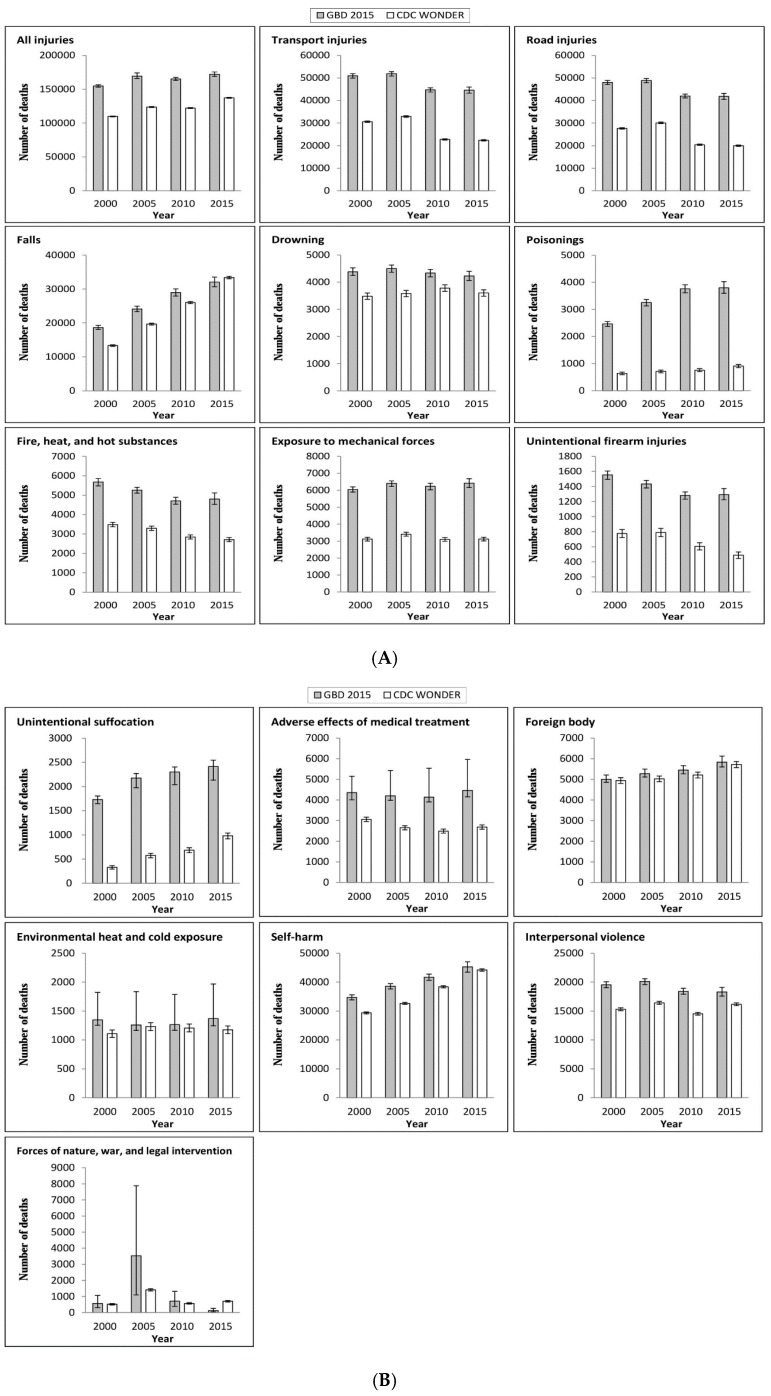
Injury deaths by cause from GBD 2015 and U.S. CDC WONDER (2000–2015). (**A**: all-cause injury and cause 1–8; **B**: cause 9–16). Note: Error bars indicate 95% uncertainty intervals from GBD 2015 estimates and 95% confidence intervals from CDC WONDER, respectively.

As shown in Figure 1 and Table 2, using the same external cause classifications of injury, GBD 2015 produces somewhat higher injury death estimates for all injury causes in all four years, except for deaths from falls and from forces of nature, war, and legal intervention in 2015, compared to CDC WONDER. The difference in overall crude deaths across the two data sources decreased from 44,897 in 2000 to 34,877 in 2015. The largest relative differences occurred in unintentional suffocation (146–429%), poisoning (286–395%), and unintentional firearm injuries (82–164%) in the four reported years.

Differences in deaths and percent difference in deaths between the two data sources varied greatly across injury cause and over the assessment years. The largest percent differences occurred in injuries from unintentional suffocation, poisoning, unintentional firearm injuries, and road injuries.

**Figure 2:**
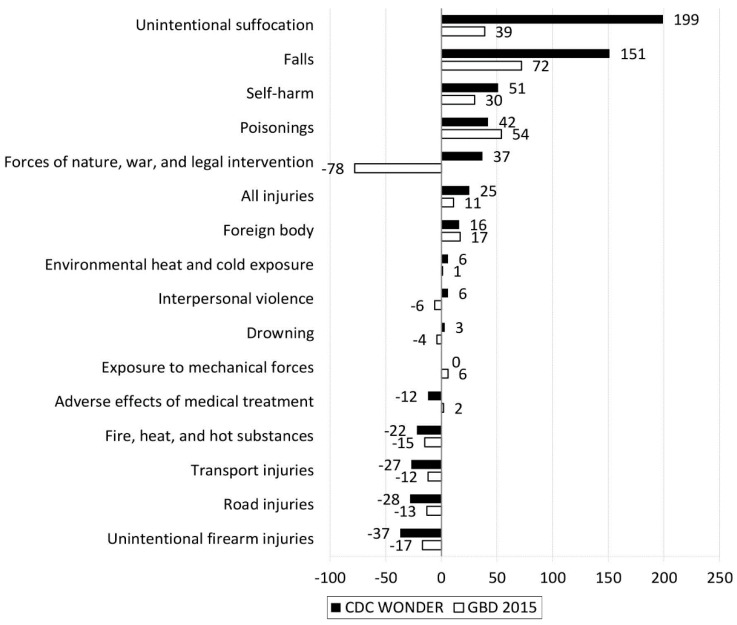
Percent changes of injury deaths between 2000 and 2015 in the United States by cause and data source. Note: Percent change in injury deaths was calculated as “(deaths in 2015 – deaths in 2000)/deaths in 2000 × 100%”.

Percent changes in crude deaths between 2000 and 2015 generally corresponded across the data sources, but moderate differences and even contrasting changes emerged for some causes (Figure 2). For example, CDC WONDER presented the greatest change in unintentional suffocation (199%), falls (151%), and self-harm (51%), but GBD 2015 showed 39%, 72%, and 30% change in crude deaths for those three injury causes, respectively.

## 4. Discussion

GBD 2015 and CDC WONDER offer somewhat inconsistent injury death numbers and change trends over time, although the inconsistency varied across injury causes and generally narrowed over time. The differences we discovered likely arise primarily from differences in estimation approaches. Unlike CDC WONDER, GBD 2015 utilizes a series of models that are applied to all 195 nations in the world (including the U.S.) in a calculated attempt to adjust for impact of unavailable and garbage codes. In the estimation process, common parameter values, such as redistribution proportions for deaths with garbage codes, are applied to all countries and for all years [3,5]. Since the methodologies of the GBD study were developed for worldwide use, some parameter values that are used in the GBD models may not precisely reflect the reality for specific countries. For example, Lee et al. applied a modified garbage code algorithm to estimate cause-specific mortality rates and years of life lost (YLLs) in South Korea and obtained a rank of five leading cause of deaths different from the estimates by the GBD 2010 update [13]. Of course, the GBD methods may also yield more accurate data since they adjust for unclear or unknown death codes.

Our results across time likely reflect changes to the GBD death redistribution algorithms, supporting the notion that GBD death redistribution algorithms yielded the differences we detected in our results. As an example, the GBD 2015 update refined the redistribution strategy for unspecified poisoning codes within the unintentional poisoning category [3], which probably led to the large observed differences in poisoning mortality between U.S. CDC WONDER and the GBD 2015 update in this study. Others have reported similar trends. For example, Wan et al. reported that different redistribution methods of garbage codes substantially affected trends in age-standardized ischemic heart disease mortality between 1991 and 2010 in China and encouraged improved redistribution of garbage codes in mortality estimation [14]. Other factors may have also contributed to the differences we detected. Injury mortality reporting practice appears to have improved recently for the US CDC WONDER dataset, for example. One recent study reported improved cause specificity of unintentional injury data for Americans aged 65+ from 1999 to 2010 in the WONDER dataset [15].

In the end, both CDC Wonder and GBD estimates have merits, both are grounded in quality science to yield reliable estimates, and it is not possible, or even appropriate, to determine which dataset is “correct”. Instead, the converging trend in the data that we detected is encouraging and suggests the actual death figures likely lie between the two estimates. Continued efforts to improve cause of death coding, reduce “garbage codes”, and accurately model missing data points are recommended to achieve the most accurate and reliable data possible.

## 5. Implications and Limitations

Our findings have important implications. Cause-specific injury mortality differences between US CDC WONDER and GBD 2015 update remain and should merit attention from injury prevention researchers and policy-makers. There is no evidence to suggest either data source is incorrect, but we do recommend interpretation of both sources and further research to interpret and eliminate differences. Supportive communication between the US CDC and the GBD study group will improve the quality of both data sources and increase their consistency.

We focused our analysis on injury-related data, but similar inconsistencies may exist for mortality from non-injury diseases in the US, and between GBD estimates and other authoritative data sources in other countries. Further validation studies will yield accurate health data for researchers and policy-makers so we can best understand trends and statuses of health outcomes among subnational, national, and global populations.

This study was limited by focusing on injury mortality estimates for the US only. The findings cannot necessarily be generalized to non-injury diseases, non-fatal statistics, or other countries. Without access to U.S. death certificate records and complete medical records, or to details of GBD data adjustment methodology, we cannot explain with confidence the precise reasons for the observed mortality differences between U.S. CDC WONDER and the GBD 2015 update. In addition, other countries with online data, such as the United Kingdom, are omitted from our analysis.

## 6. Conclusions

In conclusion, the GBD 2015 and CDC WONDER datasets present somewhat different injury death numbers for the U.S. These differences could potentially impact policy-making and intervention development, warranting further studies to interpret the inconsistencies and develop estimation approaches that continue to increase consistency between the two data sources.

## Figures and Tables

**Table 1:** Injury causes and associated codes from the 10th International Classification of Diseases (ICD-10) included in the GBD 2015 estimates.

Injury Cause	ICD10 Code
All Injuries	V00-V86.99, V87.2-V87.3, V88.2-V88.3, V90-V98.8, W00-W46.2, W49-W62.9, W64-W70.9, W73-W75.9, W77-W81.9, W83-W94.9, W97.9, W99-X06.9, X08-X39.9, X46-X47, X47.1-X47.8, X48-X48.9, X50-X54.9, X57-X58.9, X60-Y08.9, Y35-Y84.9, Y87.0-Y87.1, Y88-Y88.3, Y89.0-Y89.1
Transport injuries	V00-V86.99, V87.2-V87.3, V88.2-V88.3, V90-V98.8
Road injuries	V01-V04.99, V06-V80.929, V82-V82.9, V87.2-V87.3
Falls	W00-W19.9
Drowning	W65-W70.9, W73-W74.9
Fire, heat, and hot substances	X00-X06.9, X08-X19.9
Poisonings	X46-X47, X47.1-X47.8, X48-X48.9
Exposure to mechanical forces	W20-W38.9, W40-W43.9, W45.0-W45.2, W46-W46.2, W49-W52, W75-W75.9
Unintentional firearm injuries	W32-W34.9
Unintentional suffocation	W75-W75.9
Adverse effects of medical treatment	Y38.9-Y84.9, Y88-Y88.3
Animal contact	W52.0-W62.9, W64-W64.9, X20-X29.9
Foreign body	W44-W45, W45.3-W45.9, W78-W80.9, W83-W84.9
Environmental heat and cold exposure	W88-W94.9, W97.9, W99-W99.9, X30-X32.9, X39-X39.9
Self-harm	X60-X84.9, Y87.0
Interpersonal violence	X85-Y08.9, Y87.1
Forces of nature, war, and legal intervention	X33-X38.9, Y35-Y38.893, Y89.0-Y89.1

The following three indicators were used to assess the difference between GBD 2015 and CDC WONDER: (a) difference in deaths were calculated as “(deaths from GBD 2015 − deaths from CDC WONDER)”; (b) percent difference in deaths was calculated as “(deaths from GBD 2015 − deaths from CDC WONDER)/deaths from CDC WONDER × 100%”. (c) Percent change in injury deaths was calculated as “(deaths in 2015 − deaths in 2000)/deaths in 2000 × 100%”.

**Table 2:** Difference in injury deaths from the GBD 2015 and CDC WONDER (U.S., 2000–2015).

Cause	Difference in Deaths				Percent Difference in Deaths (%)			
	2000	2005	2010	2015	2000	2005	2010	2015
All injuries	44,897	45,534	42,965	34,877	41	37	35	25
Transport injuries	20,335	18,949	21,955	22,248	66	58	96	99
Road injuries	20,306	18,742	21,561	21,809	73	62	106	109
Falls	5297	4421	2930	−1304	40	22	11	−4
Drowning	900	917	553	624	26	26	15	17
Fire, heat, and hot substances	2190	1960	1867	2091	63	59	66	77
Poisonings	1827	2543	3000	2885	286	358	395	318
Exposure to mechanical forces	2929	3002	3137	3302	94	88	101	106
Unintentional firearm injuries	778	645	675	803	100	82	111	164
Unintentional suffocation	1404	1603	1618	1434	429	280	237	146
Adverse effects of medical treatment	1298	1547	1647	1773	42	58	66	66
Foreign body	60	250	241	117	1	5	5	2
Environmental heat and cold exposure	240	29	57	194	22	2	5	16
Self-harm	5357	5939	3342	1081	18	18	9	2
Interpersonal violence	4241	3697	3876	2141	28	23	27	13
Forces of nature, war, and legal intervention	51	2113	142	−581	10	150	25	−82

Note: Difference in deaths was calculated as “(deaths from GBD 2015 − deaths from CDC WONDER)”. Percent difference in deaths was calculated as “(deaths from GBD 2015 − deaths from CDC WONDER)/deaths from CDC WONDER × 100%”.
